# Acceptability of medical male circumcision in the traditionally circumcising communities in Northern Tanzania

**DOI:** 10.1186/1471-2458-11-373

**Published:** 2011-05-23

**Authors:** Mwita Wambura, Joseph R Mwanga, Jacklin F Mosha, Gerry Mshana, Frank Mosha, John Changalucha

**Affiliations:** 1 National Institute for Medical Research, P.O. Box 1462, Mwanza, Tanzania

**Keywords:** Medical Male Circumcision, HIV infection, Traditional Circumcision, Tanzania, Africa

## Abstract

**Background:**

Data from traditionally circumcising communities show that non-circumcised males and those circumcised in the medical settings are stigmatised. This is because traditional circumcision embodies local notions of bravery as anaesthetics are not used. This study was conducted to assess the acceptability of safe medical circumcision before the onset of sexual activity for HIV infection risk reduction in a traditionally circumcising community in Tanzania.

**Methods:**

A cross-sectional study was conducted among males and females aged 18-44 years in traditionally circumcising communities of Tarime District in Mara Region, North-eastern Tanzania. A face-to-face questionnaire was administered to females to collect information on the attitudes of women towards circumcision and the preferred age for circumcision. A similar questionnaire was administered to males to collect information on socio-demographic, preferred age for circumcision, factors influencing circumcision, client satisfaction, complications and beliefs surrounding the practice.

**Results:**

Results were available for 170 males and 189 females. Of the males, 168 (98.8%) were circumcised and 61 (36.3%) of those circumcised had the procedure done in the medical setting. Of those interviewed, 165 (97.1%) males and 179 (94.7%) females supported medical male circumcision for their sons. Of these, 107 (64.8%) males and 130 (72.6%) females preferred prepubertal medical male circumcision (12 years or less). Preference for prepubertal circumcision was significantly associated with non-Kurya ethnic group, circumcision in the medical setting and residence in urban areas for males in the adjusted analysis. For females, preference for prepubertal circumcision was significantly associated non-Kurya ethnic group and being born in urban areas in the adjusted analysis.

**Conclusions:**

There is a shift of preference from traditional male circumcision to medical male circumcision in this traditionally circumcising population. However, this preference has not changed the circumcision practices in the communities because of the community social pressure. Male circumcision national program should take advantage of this preference of medical male circumcision by introducing safe and affordable circumcision services and mobilising communities in a culturally sensitive manner to take up circumcision services.

## Background

HIV infection and AIDS remain an important public health problem in Tanzania and in other sub Saharan countries [1,2]. Promoting effective interventions that prevent new infection and control the epidemic is a priority. Circumcision has been shown to have a protective effect against HIV infection acquisition in men in several observational studies [3-7] and in three randomized controlled trials (RCT) conducted in sub-Saharan Africa [8-10].

Tanzania and other countries of sub-Saharan Africa with high prevalence of HIV infection are at different stages of scaling-up safe male circumcision as an additional, and important, strategy to prevent HIV transmission. However, a serious concern is whether male circumcision in a medical setting will be acceptable in a population where traditional male circumcision has been practiced for centuries.

Findings from a qualitative study conducted in the study areas showed that traditional male circumcision is an important stage of initiation for boys aged between 10 and 18 years [11]. The practice is done as a rite of passage from infancy into adulthood. During the circumcision procedure, neither anaesthesia nor suturing of the wound is allowed. This demonstrates the courage and bravery of the initiate as it is believed that pain prepares the individual to take social responsibilities. Initiates are circumcised outside the medical setting by traditional circumcisers without properly sterilized equipment which may lead to severe and life threatening complications[11]. Non-circumcised males and those circumcised in the formal health sector are labelled and stigmatised against because customs reward bravery [11,12]. Clan leaders oversee the planning and the implementation of the circumcision practices. Rituals are conducted by the clan leaders to appease the spirits of the ancestors before the start of the circumcision season[11]

Circumcision conducted in medical settings with full adherence to medical ethics is termed medical male circumcision. Medical male circumcision is provided confidentially, without coercion and with counselling to initiates beforehand. However, the differences between traditional and medical circumcision procedures could affect the acceptability of medical male circumcision in traditionally circumcising populations. These include variations in amount of foreskin left, the absence of traditional blessings, and bypassing clan leaders in the community [13,14]. The context and meaning of the procedure may affect community perception of service providers and the equipment used [13,14].

Traditional circumcision has also been shown to be associated with high rates of complications and adverse events [12,15-17]. Data from adolescents and young men from a traditionally circumcising community in Kenya shows that the proportion who were sexually active before they were circumcised, was greater among those who were circumcised traditionally (63.1%) than those circumcised medically (35.5%) [15]. Therefore, a safe circumcision procedure for males prior to sexual debut may produce a greater protective effect, as it is performed before initiation of sexual activities. Data from the demographic health survey in Tanzania shows that the median sexual debut age was 18 years in 2003/04 [18]. These circumstances provided the ideal opportunity to assess the acceptability of medical male circumcision performed before initiation of sexual activities for HIV prevention in Tarime district, Northern Tanzania where males are mostly circumcised as young adults in the traditional settings.

## Methods

A cross-sectional and descriptive study was conducted as part of the situation analysis for male circumcision in Tanzania to assess the context, extent and pattern of circumcision practices in selected areas of Tanzania and to assess the acceptability and feasibility of carrying out safe circumcision services in the health facilities. The study was undertaken to inform planned scaling up of circumcision services in Tanzania.

To assess cultural issues associated with circumcision in a traditionally circumcising population, Mara Region was selected to participate in the study, while Mbeya and Kagera Regions were selected to assess attitudes of traditionally non-circumcising populations towards circumcision. The two regions were selected in traditionally non-circumcising population because the prevalence of HIV infection is higher in traditionally non-circumcising population compared to circumcising populations [18].

Prior to the study, an initial visit was done to explain the rationale and objectives of the survey to regional, district and community authorities. During this visit, list of districts that traditionally circumcise in Mara Region was generated. From this list, Tarime district was selected randomly to participate in the study and a list of all health facilities in the district was generated. Health facilities were then stratified by locality (rural, roadside, and urban), and one health centre was randomly selected from each stratum. A list of sub-villages located in the service area of the health centre was compiled and 3 sub-villages (9 in total) were randomly selected into the study with probability proportional to the sub-village size. For each selected sub-village, a list of all household heads was compiled by the sub-village leader and one household was selected randomly. Nine other households were selected on the basis of being nearest to the household under survey. All *defacto *household members who were aged 18-44 years and who consented to participate in the study were included in the study.

Private and confidential interviews were conducted at the central place for each present, eligible and consenting individual. All interviews were conducted in *Swahili *(the national language) by same sex interviewers using two structured questionnaires, one for females and another for males. The male questionnaire contained questions on socio-demographic, preferred age for circumcision, circumcision status, barriers to circumcision, factors influencing circumcision, client satisfaction, complications and beliefs surrounding the practice. The female questionnaire contained questions about participants' opinion on sexual pleasure, their attitudes towards circumcision and preferred age for circumcision. Non-attendees were traced in their homes, if found they were interviewed. The circumcision status of all male participants was ascertained via physical examination, conducted by a medical doctor.

Soap, worthy 0.13 US$, was provided as compensation for the participants' time, with an additional five pieces of soap, worth 0.67 US$, being given to the head of household. There was no monetary payment for participating in the study. Free treatment of sexually transmitted infections (STI) was given to any male observed with symptoms or signs of an STI, and any female reporting STI symptoms. Field conditions did not permit genital examination for females.

The ethical clearance for the study was obtained from Medical Research Coordinating Committee (MRCC) of the National Institute for Medical Research, Tanzania. This protocol was also approved by Columbia University Ethics Committee and the ethics committee of the Centres for Disease Control and Prevention (CDC) and Global AIDS Program Associate Director of Science (GAP/ADS).

### Statistical Analysis

The aim of this paper is to describe the factors that influence medical male circumcision practices among males and females in a traditionally circumcising culture.

The investigators aimed to accurately determine the proportion of males and females who preferred prepubertal circumcision in the medical setting. The preference for prepubertal circumcision in the medical setting was expected to range between 10-30% [19]. If the total population of adult men and women in Tarime district is estimated at 300,000, a sample size of 250 for this survey would be sufficient to determine preference for prepubertal circumcision in the medical setting with a standard error of +/- 2.5% level. To obtain a representative sample from the study communities, respondents were sampled from rural, roadside and urban communities. Intra-cluster correlation was estimated to be 30% and the sample size was increased to 330 participants.

Logistic regression model was used to investigate the association between the preference for prepubertal circumcision in the medical setting, as an outcome variable, and socio-demographic factors as independent variables. Preference was defined as any positive attitude by respondents towards prepubertal circumcision in a medical setting. Crude and adjusted odds ratios (OR) and 95% confidence intervals (95% CI) were obtained, and p-values taken from the log likelihood ratio test (LRT).

Life tables were created and used to compare the circumcision rates among males circumcised in the clinical and traditional settings. Age at circumcision (time of exposure) was calculated using time elapsed from birth to the reported date of circumcision. Cumulative frequency of males circumcised in the clinical and traditional settings were computed by age of circumcision, with circumcised males exiting at the time of circumcision. All males were censored at age 28 years. The log rank test was used to compare the age at circumcision between the practice of medical and traditional circumcision.

## Results

### Attendance

Overall 359 respondents were interviewed (Table 1 and 2). The response rate was 99.7%. Most participants were aged between 18 and 34 years (74.1% males, 80.9% females), had completed primary school education (67.7% males, 67.7% females) and were born in rural areas (69.1% males, 68.6% females). The majority were married (74.7% males, 89.4% females) and Christians (90.5% males, 95.2% females).

**Table 1:** Factors predicting preference for child circumcisions among Men

Factor	Total N = 170 (%)	Prepubertal medical Circumcision n = 107 (%)	Crude OR (95% CI)	Adjusted OR (95% CI)
**Age group**				
18-24 years	50 (29.4)	30 (60.0)	1 *(P = 0.39)*	1 *(P = 0.26)*
25-34 years	76 (44.7)	52 (68.4)	1.44 (0.69-3.04)	1.65 (0.70-3.85)
35-44 years	44 (25.9)	25 (56.8)	0.88 (0.39-2.00)	0.80 (0.29-2.20)
				
**Education**				
None or Incomplete Primary	29 (17.1)	18 (62.1)	1 *(P = 0.006)*	1 *(P = 0.049)*
Primary School	115 (67.7)	66 (57.4)	0.82 (0.36-1.90)	0.61 (0.23-1.61)
Above Primary School	26 (15.3)	23 (88.5)	4.69 (1.13-19.3)	2.75 (0.59-12.88)
				
**Marital status**				
Married	127 (74.7)	80 (63.0)	1 *(P = 0.98)*	
Others	43 (25.3)	27 (62.8)	0.99 (0.48-2.03)	
				
**Religion †**				
Christians	153 (90.5)	98 (64.1)	1 *(P = 0.54)*	
Others	16 (9.5)	9 (56.3)	0.72 (0.25-2.04)	
				
**Tribe**				
Kurya	129 (75.9)	71 (55.0)	1 *(P < 0.001)*	1 *(P = 0.03)*
Others	41 (24.1)	36 (87.8)	5.88 (2.17-15.95)	3.12 (1.05-9.22)
				
**Present place of Birth † †**				
Rural	116 (69.1)	69 (59.5)	1 *(P = 0.10)*	
Roadside Centre	19 (11.3)	11 (57.9)	0.94 (0.35-2.50)	
Urban	33 (19.6)	26 (78.8)	2.53 (1.02-6.31)	
				
**Place of Residence †**				
Rural	69 (40.8)	36 (52.2)	1 *(P < 0.001)*	1 *(P = 0.004)*
Roadside Centre	47 (27.8)	26 (55.3)	1.13 (0.54-2.39)	1.07 (0.47-2.44)
Urban	53 (31.4)	45 (84.9)	5.16 (2.12-12.53)	4.63 (1.67-12.84)
				
**Providers for MC † †**				
Traditional	107 (63.7)	55 (51.4)	1 *(P < 0.001*)	1 *(P = 0.012)*
Medical	61 (36.3)	51 (83.6)	4.82 (2.22-10.48)	2.95 (1.24-7.01)

† 1 person had missing data; **†† **2 people had missing data;

**Table 2:** Factors predicting preference for child circumcisions among Women

Factor	Total N = 189 (%)	Prepubertal medical Circumcision n = 135 (%)	Crude OR (95% CI)	Adjusted OR (95% CI)
**Age group**				
18-24 years	80 (42.3)	52 (65.0)	1 *(P = 0.18)*	1 *(P = 0.17)*
25-34 years	73 (38.6)	54 (74.0)	1.53 (0.76-3.07)	1.53 (0.72-3.26)
35-44 years	36 (19.1)	29 (80.6)	2.23 (0.87-5.74)	2.50 (0.90-6.94)
				
**Education**				
None or Incomplete Primary	49 (25.9)	31 (63.3)	1 *(P = 0.10)*	
Primary School	128 (67.7)	93 (72.7)	1.54 (0.77-3.10)	
Above Primary School	12 (6.4)	11 (91.7)	6.39 (0.76-53.63)	
				
**Marital status**				
Married	169 (89.4)	119 (70.4)	1 *(P = 0.35)*	
Others	20 (10.6)	16 (80.0)	1.68 (0.54-5.28)	
				
**Religion †**				
Christians	179 (95.2)	127 (71.0)	1 *(P = 0.20)*	
Others	9 (4.8)	8 (88.9)	3.28 (0.40-26.85)	
				
**Tribe**				
Kurya	128 (67.7)	84 (65.6)	1 *(P = 0.008)*	1 *(P = 0.034)*
Others	61 (32.3)	51 (83.6)	2.67 (1.24-5.77)	2.31 (1.03-5.17)
				
**Place of Birth †**				
Rural	129 (68.6)	84 (65.1)	1 *(P < 0.001)*	1 *(P = 0.001)*
Roadside Centre	25 (13.3)	17 (68.0)	1.14 (0.46-2.84)	1.17 (0.46-3.02)
Urban	34 (18.1)	33 (97.1)	17.7 (2.34-133.5)	19.34 (2.52-148.25)
				
**Present place of Residence**				
Rural	64 (33.9)	37 (57.8)	1 *(P = 0.002)*	1 *(P = 0.16)*
Roadside Centre	61 (32.3)	43 (70.5)	1.74 (0.83-3.66)	1.68 (0.74-3.84)
Urban	64 (33.9)	55 (85.9)	4.46 (1.88-10.56)	2.35 (0.92-5.98)

† 1 people had missing data;

### Male Circumcision Practices

**Figure 1:**
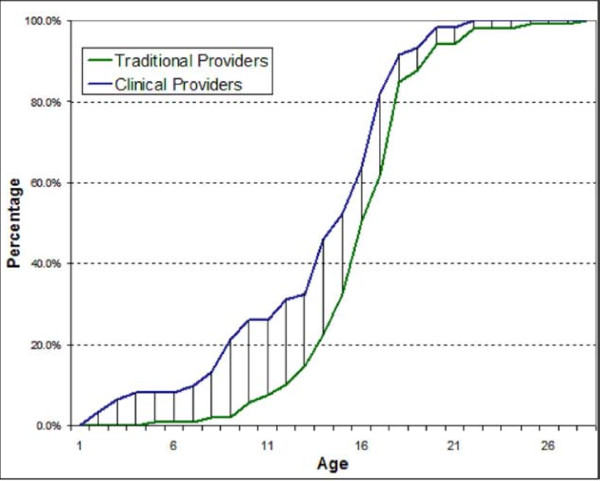
Cumulative percentage of age at circumcision by providers

Of the 359 interviewees, 170 (47.4%) were males. Of the males, 168 (98.8%) were circumcised. Sixty one (36.3%) of the circumcised males had their procedure done in medical settings while 107 (63.7%) were circumcised in the traditional settings. The age at circumcision varied significantly with the provider of the procedure (Figure 1). Of those circumcised in the medical setting, 16/61 (26.2%) were circumcised by age 10 years, whilst only 6/107 (5.6%) were circumcised by the age of 10 years in traditional settings. Males circumcised in the medical setting were circumcised at a younger age compared to those circumcised in the traditional setting (log Rank test for equality of survival functions = 9.5, P = 0.002). Cost for circumcision procedures done in the medical setting was higher compared to the cost for procedures done in the traditional setting (Wilcoxon rank sum test = 2.9, P = 0.003). The median costs for circumcision in a traditional setting were 3 US$ while in the medical setting were 5 US$.

Males who participated in the study were examined by the clinician to validate their reported circumcision status. Of those who were circumcised based on the clinician assessment (n = 144), 100% correctly reported that they were circumcised. Similarly, of those who were not circumcised based on the clinician assessment (n = 5), 2 (40.0%) correctly reported that they were not circumcised. Four males who reported to be circumcised were assessed to be partially circumcised by the clinician. Of these, one was circumcised traditionally while three were circumcised in medical settings. Seventeen males refused to be examined by the clinician (Table 3).

**Table 3:** Self-reported versus Clinician Assessment of Circumcision Status

Self-Reported Status	Clinician Assessment				
	
	Circumcised	Not Circumcised	Partially Circumcised	Refused Examination	Total
Circumcised	144 (100.0)	3 (60.0)	4 (100.0)	17 (100.0)	168 (98.8)
Not Circumcised	0 (-)	2 (40.0)	0 (-)	0 (-)	2 (1.2)
Total	144 (100.0)	5 (100.0)	4 (100.0)	17 (100.0)	170 (100.0)

### Medical Male Circumcision prior to Sexual Debut

**Figure 2:**
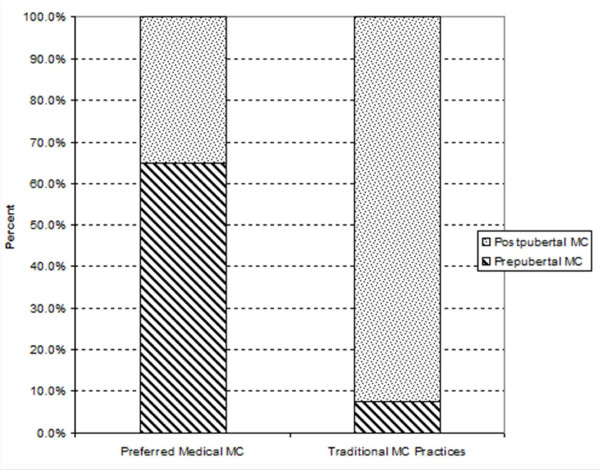
Preferred medical and current traditional practices by age at circumcision

Of the 170 male and 189 female interviewees, 165 (97.1%) males and 179 (94.7%) females supported medical male circumcision for their sons (Table 4). Of these, 107 (64.8%) males and 130 (72.6%) females preferred prepubertal circumcision (12 years or less) while 58 (35.2%) males and 49 (27.4%) females preferred postpubertal circumcision (above 12 years) in the medical setting. Reasons for prepubertal circumcision in the medical setting were the wound healing faster, bleeding and pain is less for prepubertal compared to postpubertal circumcision and there is no loss of production time during the wound healing period. Figure 2 shows the preferred age at circumcision in the medical settings and the existing circumcision practices in Tarime District, Northern Tanzania.

**Table 4:** Attitudes towards Medical and Traditional Male Circumcision by Sex

Factor	Male n = 170	Female n = 189
**Where they would like their sons to be circumcised ¶**		
Medical male circumcision (MMC)	165 (97.1%)	179 (94.7%)
Traditional male circumcision	27 (15.9%)	33 (17.5%)
		
**Of those who chose MMC, age at circumcision**		
Prepubertal circumcision	107 (64.8%)	130 (72.6%)
Postpubertal circumcision	58 (35.2%)	49 (27.4%)
		
**Attitudes in favour of Traditional Circumcision**		
Keep the tradition	113 (66.5%)	120 (63.5%)
Sign of maturity	38 (22.4%)	54 (28.6%)
Others	20 (11.2%)	15 (7.9%)
		
**Attitudes in favour of Medical male Circumcision**		
Prior to STIs	61 (35.9%)	50 (29.4%)
Avoid Pain	47 (27.6%)	48 (28.2%)
Healing is faster	18 (10.6%)	31 (18.2%)
No Bleeding	33 (19.4%)	49 (28.8%)
Others	11 (6.5%)	11 (6.5%)
		
**Attitudes against Traditional Circumcision**		
Bleeding	80 (47.1%)	76 (40.2%)
Delayed Healing	60 (35.3%)	59 (31.2%)
Others	30 (17.6%)	54 (28.6%)
		
**Attitudes against Medical Circumcision**		
Peer Pressure	62 (36.4%)	69 (36.5%)
Respect	59 (34.7%)	61 (32.3%)
Others	49 (28.8%)	59 (31.2%)

¶ Not mutually exclusive

Among males (Table 1), preference for prepubertal circumcision in the medical setting was significantly associated with above primary school education, non-Kurya ethnic tribe, urban residence and uptake of medical male circumcision in the crude analysis. Among females, significant predictors for prepubertal circumcision in the medical setting in the crude analysis were non-Kurya ethnic tribe, urban birth and residence (Table 2). In the adjusted analysis, non-Kurya ethnic tribe, urban residence and uptake of medical male circumcision remained significantly associated with preference for prepubertal circumcision among males in the medical setting while education was marginally associated with preference for prepubertal circumcision among males in the medical setting. Among females, non-Kurya ethnic tribe and urban birth remained significant predictors for prepubertal circumcision in the adjusted analysis.

## Discussion

This study has shown that, in this traditionally circumcising population, most respondents supported medical male circumcision for their sons. However, the practice in this community was different as 63.7% of the males were circumcised in the traditional settings. This observation is supported by findings from the qualitative study done in the same communities concurrently with this study (13). The discrepancy between preference and practices may be explained by the community social pressure on traditional circumcision [11,12], and most people had been circumcised several years ago. People are aware of the problems of male circumcision in the traditional settings and prefer changing to circumcision in medical setting, however due to social pressure, the uptake of medical male circumcision is still low. Community mobilisation and open dialogue at the community level on cultural beliefs about circumcision and provision of safe male circumcision services in clinical settings in a culturally sensitive manner will be crucial in helping parents in this community to take their sons for circumcision services in the medical settings. Such engagement has already been successful in the traditionally circumcising Babukusu Community in Western Kenya where following such negotiations in 2002 when only 1700 boys were circumcised in the medical settings, the situation had changed by 2006, when16,000 boys were circumcised in the medical setting [20].

Males circumcised in the clinical settings were younger than those circumcised in the traditional settings because traditional circumcision initiates a boy into manhood and prepares the individual to take social responsibilities. To preserve the socio-cultural importance of the circumcision process, while improving safety and acceptability of the circumcision procedure and enhancing health education to the communities, clinical providers will need to collaborate with the community leaders. Such collaboration has been reported in Kenya [21,22]. In the South African Eastern Cape region, medical circumcision followed by traditional initiation into manhood still lacks acceptability as 70% of initiates feared being stigmatised if they are circumcised in the medical setting [23]

This study has also shown that prepubertal circumcision in the medical setting was acceptable in this population. The preference for prepubertal circumcision in the medical setting was due to the notion that the wound heals faster, there is less pain and bleeding and there is no loss of production time during the wound healing period. Therefore, it is important that the national male circumcision programme meets the minimum quality of care for males who accepts circumcision services in the medical settings to minimise adverse events that may drive the clients away. In addition, prepubertal medical circumcision may carry a lower risk of a compensatory increase in sexual behaviour following circumcision. It also reduces the risk of HIV infection acquisition resulting from resumption to sex prior to proper wound healing and provides an ample time for keratin to develop before initiation of sexual activities [24].

Medical male circumcision procedure was costly compared to traditional circumcision procedure. Cost for circumcision is likely to be barrier for circumcision in this community as poor people especially in rural areas are less likely to afford to pay circumcision in the medical setting compared to traditional setting. Cost for circumcision was a major factor motivating preference for clinical circumcision in Kenya [15].

Using self reported circumcision status was very sensitive but not very specific compared to clinician assessed circumcision status. The lack of specificity using self reported circumcision status was due to social desirability bias caused by stigma and discrimination for non-circumcised males [12]. We recommend studies conducted in traditionally circumcising communities to validate the self-reported circumcision status.

There are several limitations to our study; age at circumcision and cost paid for the procedure were collected retrospectively. Males circumcised in the traditional setting may have found it difficult to value the money paid for the procedure if the payment was done in kind and therefore underestimate the cost for the procedure. Likewise, males circumcised in the clinical setting are likely to be unaware of the age at circumcision if they were circumcised as infants. However, infants' circumcision is rare in this community.

## Conclusions

There is a shift of preference from traditional male circumcision to medical male circumcision in this traditionally circumcising population. This preference was due to the notion that medical male circumcisions heals faster, with no bleeding and pain and is done prior to males initiating sexual activity and therefore prior to acquiring STIs. However, this preference has not changed the circumcision practices in the communities because of the community social pressure. The national male circumcision programs should take advantage of this preference of medical male circumcision by introducing safe and affordable circumcision services and mobilising communities in a culturally sensitive manner to take up circumcision services.

Bleeding and delay of the wound to heal were cited as major reasons for this shift of preference from traditional to medical male circumcisions. As services are rolled out, if medical male circumcision is seen to be associated with adverse outcomes, this may create additional demand for traditional circumcision practices. Therefore, as the services are rolled out, the quality of care provided should be measured to determine whether the standards are being met and quality improvement methodology should be used to continuously improve the quality of circumcision care and services.

## Competing interests

The authors declare that they have no competing interests.

## Authors' contributions

MW, JRM, JFM and JC designed this study. GM, FM, MW, JRM, JFM collected and analyzed the quantitative and qualitative data while JC provided the editorial input. All authors read and approved the final manuscript.

## Pre-publication history

The pre-publication history for this paper can be accessed here:

http://www.biomedcentral.com/1471-2458/11/373/prepub
